# Percutaneous nephroscopy combined with the laser used for right kidney bullet extraction: A case report

**DOI:** 10.1097/MD.0000000000032841

**Published:** 2023-02-10

**Authors:** Baolong Wang, Lijun Yang, Jianlin Yuan, Weijun Qin, Peng Wu, Xiaojian Yang, Guangdong Hou, Ming Yu, Xue Gong, Zhicheng Xu, Jun Qin, Xuelin Gao, Shuaijun Ma, Fuli Wang

**Affiliations:** a Department of Urology, Xijing Hospital, Fourth Military Medical University, Xi’an, China; b Department of Ultrasound, Xi’an Daxing Hospital, Shaanxi University of Chinese Medicine, Xi’an, China.

**Keywords:** bullet extraction, kidney, laser, minimally invasive, percutaneous nephroscope

## Abstract

Wounds caused by firearms are intractable problems in treating war traumas and clinical management. Conventional open surgery inflicts large injury and leads to slow recovery. At the same time, most patients suffer from compound injuries with the critical condition and poor operation tolerance. Thus, it is crucial to probe into the minimally invasive surgical removal of residual kidney bullets.

**Patient concerns:**

We report a case where a bullet remained in the right renal parenchyma on the patient, with penetrating injury in his liver.

**Diagnosis:**

Obviously the patient has suffered gunshot wound with a bullet stuck in his kidney, while his liver function was impacted.

**Interventions:**

Six months after the injury, we performed the minimally-invasive procedures on the patient with percutaneous nephroscope technology and laser technology under the guidance of ultrasound localization. The bullet and ammunition granulation and scar surrounding tissue were fully removed. Intraoperative bleeding was little, while the incision was small. The patient could leave the bed and walk on the 1st postoperative day. The drainage tube was removed on the 3rd postoperative day, after which the patient was discharged on the 4th postoperative day.

**Outcomes:**

The patient recovered well after surgery and was followed up for 5 years. The latest examination of his liver and kidney function was as follows: alanine aminotransferase 61IU/L, aspartate aminotransferase 33 IU/L, albumin/globulin 46.6/26.0, total bilirubin 19.1μmol/L, direct bilirubin 4.9μmol/L, indirect bilirubin 14.2μmol/L, alkaline phosphatase 111 IU/L, creatinine 57μmol/L, urea 5.16mmol/L, cystatin 0.73mg/L. The plain computed tomography scan showed a few calcifications in the liver and a patchy low-density shadow in the right kidney. It was proved that the liver and kidney function of the patient recovered well, and his living qualify has come back to the track, with no postoperative complications.

**Lessons:**

Innovative integration of percutaneous nephroscopy technology and laser was used to remove kidney foreign bodies and developed the optimal surgical plan, small trauma, fast recovery, and the treatment of kidney foreign bodies was newly explored.

## 1. Introduction

Organ firearm injury is common today, and the foreign body retainment caused by it is not only a difficult problem for treating war trauma but also a challenging topic in clinical surgery.^[1]^ The most common type of surgery is bullet retainment.

Bullet retainment after firearm injury to the kidney is a puzzle of war trauma treatment, with great hardness in clinical diagnosis and surgical operation process.^[2]^ The traditional removal of renal foreign bodies requires open or laparoscopic operations, which spawns great trauma, much bleeding and a long recovery time for patients. Meanwhile, the condition of emergency patients with firearm injuries is unstable, and their tolerance to open surgery is poor. Similarly, laparoscopic surgery necessitates establishing 3 to 4 channels to dissociate the kidney fully, and the renal artery needs to be blocked during the operation, affecting the renal function of patients. Relatively speaking, as a minimally invasive surgical method, percutaneous nephroscopy is mainly used to establish percutaneous renal channels for the treatment of upper urinary calculi, the treatment of ureteropelvic junction stricture and removal of foreign bodies, such as displaced ureteral stents.^[3]^

## 2. Case presentation

The patient, a 34-year-old male, was admitted with “right chest and abdominal trauma for six months.” The patient was injured by a firearm accidentally 6 months ago. The bullet passed through the right midclavicular line and the right 11th intercostal area, resulting in a perforation of the right lobe of the liver and damage to the right kidney. A small amount of bleeding occurred at the wound site. The patient is in stable condition at admittance with no hematuria or other symptoms. The gentleman requested to remove the bullet from his body.

**Figure 1.**
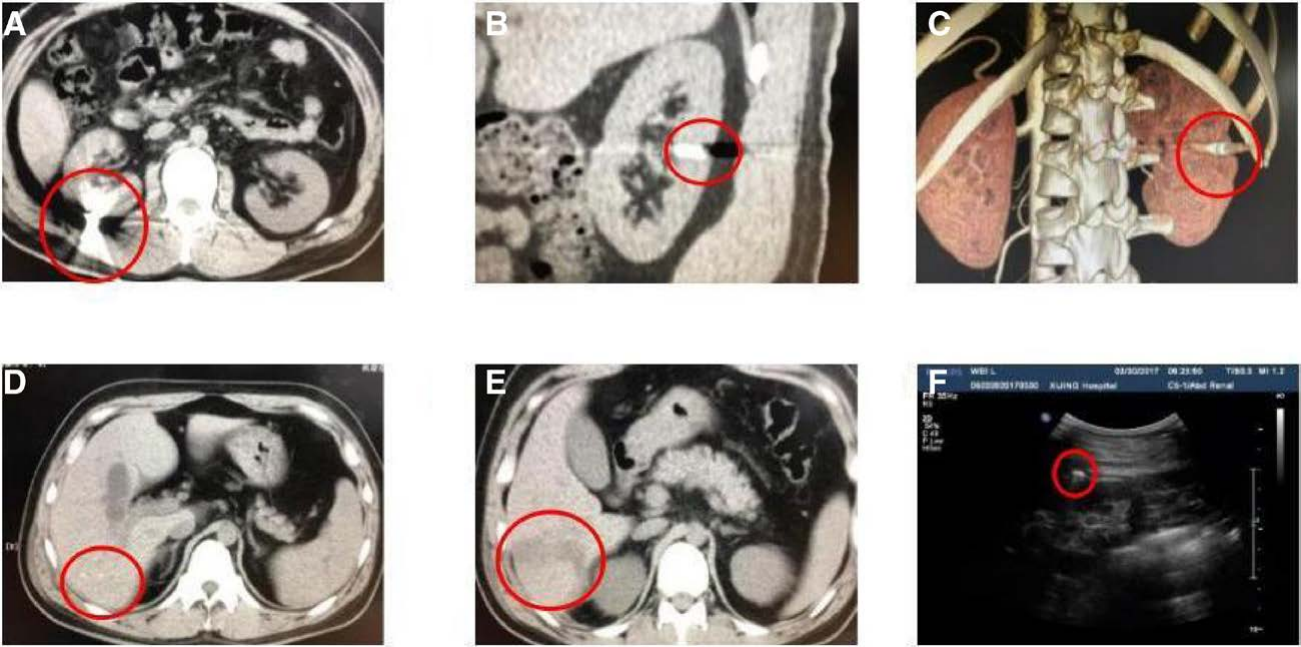
Before surgery, there was a small nodular density enhancement in the right kidney and a small calcification in the right lobe of the liver with a low-density trajectory. (A) Plain CT transverse view of right kidney. (B) Plain CT longitudinal view of right kidney. (C) Three-dimensional CT imaging of the kidney. (D) Small calcification foci in the right lobe of liver. (E) Liver low-density ballistics. (F) Right renal parenchyma ultrasonography strong echo light mass with acoustic shadow. CT = computed tomography.

Physical examination results are as follows: consciousness is clear, stable vital signs, skin sclera without yellow staining, soft abdomen, no tenderness, no rebound pain, visible scar on abdominal skin, and no lumps on waist and abdomen. There was no tenderness and percussion pain in both renal regions. Computed tomography (CT) examination showed minimal nodular increased density in the right kidney (Fig. 1A–C), small calcification in the right lobe of the liver (Fig. 1D, Figure Legends), and low-density trajectory (Fig. 1E). Preoperative ultrasound images are shown in Figure 1F. Patient’s blood lead was normal, ruling out lead poisoning from bullet retainment.

General anesthesia was carried out with the patient in a prone position. Under the guidance of ultrasound, the condition of the bullet retained was observed. Then we chose the node of the right collarbone midline and the 11th intercostal area as the puncture point. Then, the puncture with an 18G needle toward the bullet in the right kidney was finished. The needle core was not pulled out until its contact with the bullet (see Supplemental Video-S1, Supplemental Digital Content, http://links.lww.com/MD/I421, which shows the process of ultrasound-guided bullet puncture of the right renal parenchyma with an 18G puncture needle.). Through the puncture needle sheath, the guide wire was applied. Along the guide wire, the fascia expander was gradually expanded from 10F to 24F, and a 24F metal operating sheath was inserted. The nephroscope was placed in the sheath, and saline was slowly injected continuously. Most parts of the bullet were embedded in renal parenchyma with poor mobility. The 1470 laser fully dissociated the granulation tissue and scars around the bullet, and the bleeding was carefully controlled. The intraoperative field of vision was clear, and the bullet was completely removed with foreign body forceps. The operation process is presented in Supplemental Video-S2, Supplemental Digital Content, http://links.lww.com/MD/I422, which recorded the process that under percutaneous nephroscopy, a 1470 laser was used to fully dissociate the tissue around the bullet, after which the bullet was completely removed with foreign body forceps. After careful examination, no bleeding was found on the surface of the wound. 20F drainage tube was inserted through a percutaneous channel after the surgery.

**Figure 2.**
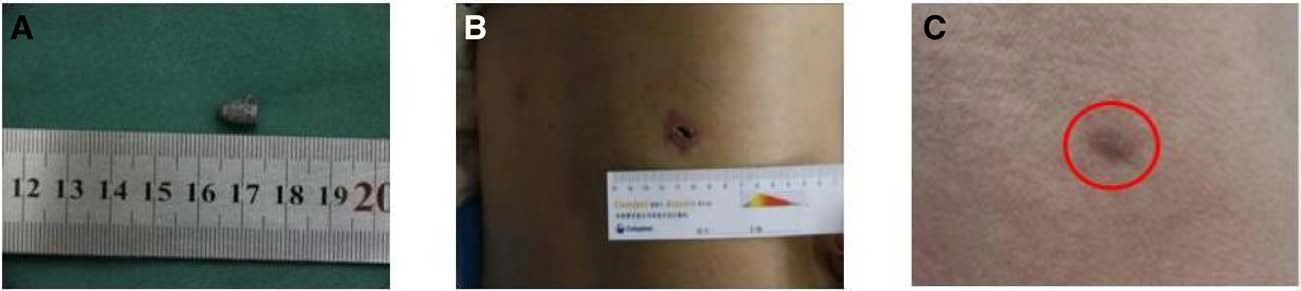
(A) The bullet was completely removed. (B) The wound was limited to 2cm. (C) The patient’s wound healing was good.

**Figure 3.**
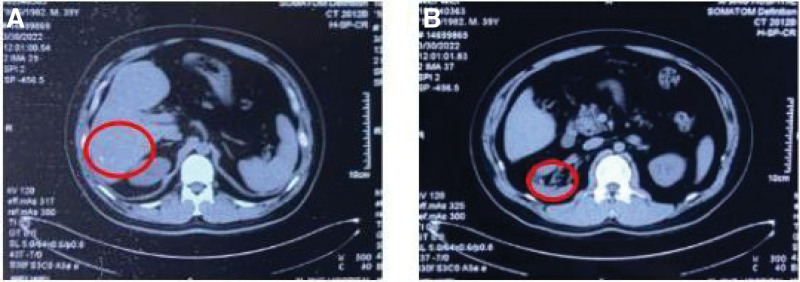
The results of a plain CT scan of the liver and kidney were followed up 5 years after the operation. (A) The results of a plain CT scan of the liver and kidney were followed up 5 years after operation. (B) Plain CT scan of the right kidney showed a patchy low-density shadow. CT = computed tomography.

The patient had no intraoperative bleeding as the bullet was completely removed (Fig. 2A). What’s impressive is that the incision was only 15 mm (Fig. 2B). The patient could get out of bed and move on the 1st postoperative day. The drainage tube was removed on the 3rd postoperative day, and the patient was discharged on the 4th postoperative day. The postoperative recovery is wonderful. The wound heals well, too (Fig. 2C). Seen from a 5-year follow-up, the results of his liver and kidney function examination are relatively satisfying: alanine aminotransferase 61IU/L, aspartate aminotransferase 33 IU/L, albumin/globulin 46.6/26.0, total bilirubin 19.1 μmol/L, direct bilirubin 4.9 μmol/L, indirect bilirubin 14.2 μmol/L, alkaline phosphatase 111 IU/L, creatinine 57 μmol/L, urea 5.16 mmol/L, and cystatin 0.73 mg/L. A plain CT scan of the liver showed calcification foci, while a plain CT scan of the right kidney showed a patchy low-density shadow (Fig. 3), with no postoperative complications.

## 3. Discussion

As far as we know, the first congeneric operation was held in 1988 by the Tulane University School of Medicine on a 33-year-old American patient.^[4]^ Twenty-four hours after the shooting, under X-ray localization, 28F percutaneous nephroscope helped take out the right renal parenchyma bullet, direct and plain. The wound was obvious when the patient was attended to. Also, the medical staff saves him with a rigid nephroscope, causing much bleeding. In addition, there was no immediate or follow-up test for blood lead. Therefore, the prognosis of the patient was unclear. In the case we faced, granulation tissue and scar appeared around the bullet 6 months after the wound, which made the bullet challenging to remove. Establishing only 1 operating channel, we successfully used percutaneous nephroscopy technology, combined with CT 3D reconstruction ultrasonic localization and precise puncture. A 1470 laser was used to fully dissociate and eliminate the bullet during the operation.

In acquiring the right position of the bullet, we chose the combined application of CT technology, 3D reconstruction and precise ultrasonic localization, which likewise assisted in the selection of puncture points. Especially, 3D modeling technology makes the whole localization progress more intuitive, accurate and reliable, providing a highly feasible modelized technique of localization.^[5]^ During the operation, a precise puncture guided by ultrasound directly reached the bullet. The combined application of CT and ultrasonic localization can effectively reduce the possibility of the injury of renal parenchyma and renal vessels during operation, which is of great significance to control or even eradicate blood loss and ensure a clear vision. The advantages of this method have been supported by other researchers. A patient with cysticercosis in India benefited from a combination of CT and ultrasound localization, which ended up with the complete removal of cyst lesions. With minimal injury and high accuracy, the patient experienced a favorable prognosis.^[6]^ Similarly, the combined application of the techniques above also has excellent effects in removing peritoneal foreign bodies, which successfully saved patients with co-infection and proved convenient in preventing infection.^[7]^

The integrated application of different minimally invasive techniques can reduce the difficulty of surgery and achieve the principle of optimization.^[8]^ First, the percutaneous nephroscopy channel adopted the 24F metal operating sheath. The bullet remaining in the right kidney was small in volume, so it could be removed through this channel. At the same time, the wound was small in size, and the incision on the skin was just 15 mm in diameter, which was quite pleasing to the eye. On the other hand, the patient suffered from retaining the bullet in the body for half a year, during which granulation and scar tissue had shaped. We were then informed by the situation of the patient that the bullet was relatively fixed, affecting the handling and hemostasis. What’s more, the difficulty we faced was far more serious than that of the case done by Tulane University School of Medicine^[4]^, whose patient was attended merely 24 hours after the gunshot. In the meantime, the bullet was not fixed, easy to remove. However, the application of the 1470 laser made the hemostasis impressively smooth and productive.^[9]^ Also, thanks to our surgeon’s accurate and pinpoint work, the probe can instantly vaporize the surrounding tissues grabbing the bullet, such as granulation tissues. No excess wounds were brought about, but a clear vision was shown, which was convenient for the subsequent removal of the bullet, further reducing complications.^[10]^ Iatrogenic injuries are found pretty commonly in this sort of operation, among which infection and bleeding are particularly prominent.^[11–13]^ However, we have effectively avoided complications with minimal intraoperative and postoperative injuries.

Lead poisoning caused by bullet retainment is also a rare but potentially life-threatening side reaction.^[14]^ The patient’s condition was stable after surgery, with normal blood lead concentration, no gross hematuria, normal red blood cell number, hemoglobin, liver functions and kidney functions, which ruled out the possibility of lead poisoning. All these results above showed that our surgical accuracy was sufficient to ensure that the bullet could be removed without contact with the tissue’s circulation during exposure and removal. The bullet can be removed intact, which is one of the keys to avoiding unnecessary complications.

Although the operation is generally successful, there are still aspects for consideration. Firstly, the patient came to our department 6 months later for treatment of the bullet in his body, before which a greater risk of lead poisoning or other complications existed, requesting a comprehensive and accurate assessment of the patient’s condition before and during surgical activity. Secondly, the feasibility of percutaneous nephroscopy combined with 1470 laser kidney bullet removal has not been fully verified due to the rarity of kindred cases. Yet, our operation certainly points to a new idea worth trying. Hopefully, the surgical protocol provided in this study can be selectively applied to subsequent emergency patients with similar conditions to verify its feasibility.

Innovative integration of percutaneous nephroscopy technology and laser was used to remove kidney foreign bodies and developed the optimal surgical plan, small trauma, fast recovery, and the treatment of kidney foreign bodies was newly explored.

## Author contributions

**Conceptualization:** Fuli Wang.

**Data curation:** Baolong Wang, Jianlin Yuan, Peng Wu, Guangdong Hou, Jun Qin, Xuelin Gao.

**Formal analysis:** Lijun Yang.

**Funding acquisition:** Fuli Wang.

**Investigation:** Lijun Yang.

**Methodology:** Weijun Qin, Ming Yu, Xue Gong, Jun Qin, Fuli Wang.

**Project administration:** Jianlin Yuan, Ming Yu, Xuelin Gao.

**Resources:** Lijun Yang, Jianlin Yuan, Peng Wu, Xiaojian Yang, Xue Gong, Shuaijun Ma.

**Software:** Xiaojian Yang, Xuelin Gao.

**Supervision:** Weijun Qin, Fuli Wang.

**Validation:** Zhicheng Xu, Jun Qin.

**Writing – original draft:** Baolong Wang, Guangdong Hou.

**Writing – review & editing:** Weijun Qin, Xiaojian Yang, Guangdong Hou, Ming Yu, Zhicheng Xu, Shuaijun Ma, Fuli Wang.

## Supplementary Material




